# Psychological resilience, job satisfaction, and anxiety symptoms in private hospital healthcare workers: a cross-sectional study

**DOI:** 10.3389/fpubh.2026.1938191

**Published:** 2026-09-04

**Authors:** Sheng Li, Yali Feng, Fei Wang, Ping Yang, Jinyu Wang, Guoai Yao

**Affiliations:** 1 The No.2 People's Hospital of Lanzhou, Lanzhou, China; 2 Lanzhou University Second Clinical Medical College, Lanzhou, China; 3 School of Public Health, Gansu University of Chinese Medicine, Lanzhou, China; 4 School of Public Health, Lanzhou University, Lanzhou, China

**Keywords:** anxiety symptoms, cross-sectional study, healthcare workers, job satisfaction, private hospitals, psychological resilience

## Abstract

**Background:**

Healthcare workers in private hospitals may experience substantial psychological challenges due to demanding work environments and organizational characteristics. Psychological resilience has been recognized as an important personal resource associated with psychological wellbeing, whereas job satisfaction represents a key motivational factor related to occupational functioning. However, the relationships among psychological resilience, job satisfaction, and anxiety symptoms among healthcare workers in private hospitals remain insufficiently understood. This study aimed to examine the associations between psychological resilience, job satisfaction, and anxiety symptoms, and to explore whether job satisfaction statistically explains the association between psychological resilience and anxiety symptoms.

**Methods:**

A multicenter cross-sectional survey was conducted among healthcare workers from 10 private hospitals in Lanzhou, China. Participants were recruited using a convenience sampling approach. Psychological resilience, job satisfaction, and anxiety symptoms were assessed using the Connor–Davidson Resilience Scale (CD-RISC), a newly developed 13-item job satisfaction scale, and the Self-Rating Anxiety Scale (SAS), respectively. Descriptive analyses, correlation analyses, multiple linear regression analyses, and mediation analyses using bootstrap methods were performed to examine the associations among study variables.

**Results:**

A total of 1,263 healthcare workers were included in the analysis. Psychological resilience was positively associated with job satisfaction and negatively associated with anxiety symptom scores (*P* < 0.001). Job satisfaction was also negatively associated with anxiety symptom scores (*P* < 0.001). Mediation analysis indicated that job satisfaction partially accounted for the association between psychological resilience and anxiety symptoms. Specifically, the indirect association through job satisfaction accounted for part of the association between psychological resilience and anxiety symptom scores.

**Conclusions:**

Psychological resilience and job satisfaction were significantly associated with anxiety symptoms among healthcare workers in private hospitals. These findings highlight the potential relevance of personal psychological resources and job-related experiences in understanding healthcare workers' psychological wellbeing. However, longitudinal studies are needed to further clarify the directionality and causal mechanisms underlying these relationships.

## Introduction

Healthcare workers are exposed to a variety of occupational demands, including high workload, emotional labor, time pressure, and responsibility for patient outcomes (1–3). These demands may negatively affect their psychological wellbeing and contribute to symptoms of anxiety, stress, and burnout (4, 5). Anxiety symptoms among healthcare workers have attracted increasing attention because they may impair occupational functioning, reduce quality of life, and potentially influence the sustainability of healthcare services (6–8). Therefore, identifying psychological and organizational factors associated with anxiety symptoms among healthcare workers is important for developing effective occupational mental health promotion strategies (9–11).

Previous studies have identified multiple factors associated with anxiety symptoms among healthcare workers, including workload, occupational stress, organizational support, and individual psychological characteristics (12, 13). However, much of the existing literature has focused on healthcare workers in public hospitals, whereas evidence regarding healthcare workers in private hospitals remains relatively limited (14). Private hospitals represent an important component of healthcare systems in many countries, yet their organizational environments, resource availability, and employment structures may differ from those of public institutions. Understanding psychological factors associated with anxiety symptoms in this setting may provide additional insights into occupational mental health among healthcare workers (15, 16).

Psychological resilience has been increasingly recognized as an important personal resource that enables individuals to adapt effectively to stressful circumstances. According to the Job Demands–Resources (JD-R) model, personal resources can influence employees' perceptions of work demands and contribute to positive psychological outcomes (17, 18). From this perspective, psychological resilience may represent an internal resource that helps healthcare workers maintain psychological balance when facing occupational challenges (19, 20). Previous research has suggested that individuals with higher resilience tend to report fewer psychological distress symptoms; however, the mechanisms underlying this association remain insufficiently explored (21–23).

Job satisfaction is another important factor related to healthcare workers' psychological wellbeing. Within the JD-R framework, motivational factors and positive work experiences may enhance employees' engagement and psychological functioning. Higher job satisfaction has been associated with better mental health outcomes, whereas dissatisfaction may increase vulnerability to psychological distress. In addition, Self-Determination Theory suggests that fulfillment of basic psychological needs in the workplace may contribute to positive emotional experiences and occupational functioning. Therefore, job satisfaction may represent a potential pathway linking individual psychological resources and anxiety symptoms.

Although psychological resilience and job satisfaction have each been linked to healthcare workers' psychological outcomes, few studies have examined their potential interplay in relation to anxiety symptoms. Existing studies have often investigated isolated predictors of psychological distress, providing limited understanding of how personal resources and workplace-related factors may jointly contribute to healthcare workers' mental health. Integrating these theoretical perspectives, this study conceptualizes psychological resilience as an internal resource, job satisfaction as a motivational and organizationally influenced psychological outcome, and anxiety symptoms as an indicator of impaired psychological wellbeing. This integrated framework provides a theoretical basis for examining whether job satisfaction mediates the association between psychological resilience and anxiety symptoms among healthcare workers in private hospitals.

To provide a contextually grounded theoretical explanation for the proposed mediation framework, this study integrates Conservation of Resources (COR) theory, the Job Demands–Resources (JD-R) model, and Self-Determination Theory (SDT). These perspectives are complementary rather than independent, as they address different but interconnected levels of the proposed relationship. Specifically, COR theory explains how individuals preserve and mobilize psychological resources when facing stress and resource loss; the JD-R model situates these personal resources within the demands and resources of the occupational environment; and SDT further explains the proximal motivational and psychological processes through which positive work experiences may contribute to psychological wellbeing (17–20).

From the perspective of COR theory, psychological resilience can be conceptualized as an important personal resource that helps healthcare workers acquire, preserve, and recover psychological resources when facing occupational stress and resource loss (17). COR theory proposes that individuals strive to obtain, retain, and protect valued resources, and that stress may occur when these resources are threatened or lost (17, 18). Accordingly, individuals with greater psychological resilience may be better able to cope with stressful circumstances, conserve psychological resources, and recover from adverse experiences. Previous research has suggested that resilience is associated with better psychological adaptation and may help reduce vulnerability to adverse mental health outcomes among healthcare professionals (12, 13, 15, 21). This perspective is particularly relevant to healthcare workers in the post-pandemic period, who may continue to encounter demanding workloads, changing service requirements, organizational uncertainty, and employment-related pressures (2, 6, 7). In such circumstances, psychological resilience may help individuals maintain emotional stability and reduce the psychological consequences of resource loss, thereby contributing to lower levels of anxiety symptoms. Thus, COR theory provides the individual-level resource explanation for the protective association between psychological resilience and anxiety symptoms in the proposed model.

The JD-R model extends this individual-level explanation by placing personal resources within the occupational environment. The model proposes that employee wellbeing is shaped by the interaction between job demands and job resources, while personal resources can facilitate adaptation to job demands and contribute to positive occupational functioning (18, 19). In healthcare settings, job demands may include heavy workloads, demanding service requirements, interpersonal pressures, and other occupational challenges, whereas organizational support and other workplace resources may facilitate adaptation and wellbeing (6, 9–11). These processes may be particularly relevant in private hospitals, where employment arrangements, workload pressures, organizational conditions, and available workplace resources may shape employees' experiences of their work (7, 9). Within this framework, psychological resilience can be viewed as a personal resource that helps healthcare workers adapt to occupational demands. Successful adaptation may, in turn, be associated with more positive perceptions and experiences of one's work, which may be reflected in greater job satisfaction. Therefore, the JD-R model provides the occupational-level explanation linking an individual's psychological resources to work-related experiences, while recognizing that these relationships are embedded within the organizational context rather than occurring independently of it.

SDT further clarifies the proximal psychological process through which positive work experiences may be associated with psychological wellbeing. SDT proposes that wellbeing and optimal functioning are supported by the fulfillment of three basic psychological needs: autonomy, competence, and relatedness (19, 20). In the workplace, the satisfaction of these needs is closely related to employees‘ motivation, positive functioning, and wellbeing (20, 22). Although job satisfaction is not equivalent to psychological need satisfaction, it may reflect employees' overall positive evaluation of their work experiences and the extent to which their work environment provides meaningful and psychologically rewarding experiences. Healthcare workers who are better able to cope with occupational challenges may be more likely to maintain positive work experiences and perceptions of their jobs. Conversely, positive work experiences characterized by greater perceived competence, autonomy, and relatedness may be associated with better psychological functioning and reduced psychological distress (20, 22). Thus, job satisfaction may represent a proximal work-related psychological pathway through which personal resources are associated with anxiety symptoms.

Taken together, COR theory, the JD-R model, and SDT provide a coherent and complementary theoretical explanation for the proposed mediation model in the context of Chinese private hospitals after the COVID-19 pandemic. COR theory explains the resource basis of the relationship by suggesting that psychological resilience enables individuals to preserve and mobilize psychological resources when facing occupational stress (17, 18). The JD-R model explains how these personal resources operate within a demanding and organizationally specific work environment and how successful adaptation to job demands may be reflected in more positive work experiences (18, 19). SDT then provides a motivational and psychological explanation for why positive work experiences, reflected in job satisfaction, may be associated with better psychological wellbeing (20, 22). Accordingly, the three perspectives together support the proposed pathway in which psychological resilience is associated with greater job satisfaction, which in turn is associated with fewer anxiety symptoms. This integrated framework therefore conceptualizes the mediation process as a progression from personal resource preservation (COR), to adaptation within the occupational context (JD-R), and finally to positive work-related psychological functioning (SDT), while recognizing that these associations are shaped by the organizational conditions characteristic of private hospitals in the post-pandemic period (2, 7, 9–11).

However, existing mediation studies examining the relationships between psychological resources, job satisfaction, and psychological distress have largely focused on healthcare workers in public hospitals, with relatively limited evidence from private hospital settings (6). This distinction is particularly relevant in the post-pandemic context of China, where private hospitals have experienced unique organizational challenges related to healthcare demand changes, operational pressures, workforce management, and employment uncertainty (3, 21, 22). Compared with public institutions, private hospitals often differ in employment structures, career development pathways, organizational management styles, and perceived job security, which may influence how healthcare workers utilize personal psychological resources and experience workplace satisfaction (8).

This study addresses several important gaps in the existing literature. First, it focuses specifically on healthcare workers in Chinese private hospitals, a population that has received substantially less attention than healthcare workers in public hospitals, particularly in the post-pandemic period. Second, rather than examining psychological resilience, job satisfaction, and anxiety symptoms as independent correlates, this study tests whether job satisfaction statistically accounts for part of the association between psychological resilience and anxiety symptoms, thereby providing a more mechanism-oriented understanding of their interrelationships. Third, by integrating individual psychological resources with work-related experiences within the specific organizational context of private hospitals, the study applies the complementary perspectives of COR theory, the JD-R model, and SDT to explain how personal resources and occupational experiences may be linked to anxiety symptoms. Together, these features provide context-specific evidence that extends existing research beyond predominantly public-hospital settings and contributes to a more comprehensive understanding of occupational mental health among healthcare workers in Chinese private hospitals.

This study aimed to investigate the associations among psychological resilience, job satisfaction, and anxiety symptoms among healthcare workers in private hospitals. Specifically, we examined whether psychological resilience was associated with anxiety symptoms and whether job satisfaction statistically accounted for part of this association. Based on the theoretical framework and previous evidence, the following hypotheses were proposed: H1: Psychological resilience is negatively associated with anxiety symptoms among healthcare workers. H2: Psychological resilience is positively associated with job satisfaction among healthcare workers. H3: Job satisfaction is negatively associated with anxiety symptoms among healthcare workers. H4: Job satisfaction statistically accounts for part of the association between psychological resilience and anxiety symptoms among healthcare workers.

## Methods

### Study design and participants

A multicenter cross-sectional study was conducted among healthcare workers from healthcare workers were recruited from 10 private hospitals in Lanzhou, China, using a convenience sampling approach. The study was designed to investigate the associations among psychological resilience, job satisfaction, and anxiety symptoms among healthcare workers. The participating hospitals were general private medical institutions providing routine clinical services, and none were tertiary public hospitals. Hospitals were selected using a convenience sampling approach based on willingness to participate and accessibility for questionnaire administration. Hospital administrators assisted with questionnaire distribution; however, they were not involved in participant selection, data interpretation, or statistical analysis. Eligible participants were healthcare workers who had worked in the participating hospitals for at least six months and voluntarily agreed to participate. A total of 1,263 valid questionnaires were collected and included in the final analysis.

The study protocol was reviewed and approved by the Ethics Committee of Lanzhou Second People's Hospital (Approval No. KY20250500025). All procedures were conducted in accordance with the ethical principles of the Declaration of Helsinki.

### Measurements

#### Demographic characteristics

Demographic information was collected using a self-administered questionnaire specifically developed for this study. Variables included sex, age, ethnicity, occupation, educational level, years of employment, professional title, and monthly income. In addition, occupational exposure and sleep-related information were collected, including needlestick injuries, sharp injuries, occupational infections, daily sleep duration, sleep initiation difficulties, and factors affecting sleep quality.

#### Job satisfaction

Job satisfaction was assessed using a 13-item scale developed by the research team based on previous literature regarding healthcare workers' occupational attitudes, organizational experiences, and work-related wellbeing. The initial item pool was generated through a comprehensive review of relevant studies and was subsequently refined through expert consultation. Experts in healthcare management and occupational health reviewed the items for relevance, clarity, and applicability to healthcare workers in private hospitals. Each item was rated using a 5-point Likert scale, with higher scores indicating higher levels of job satisfaction. Before the formal survey, the preliminary scale was evaluated through exploratory factor analysis (EFA) to examine its construct validity. The psychometric properties of the scale are further presented in the Results section.

#### Anxiety symptoms

Occupational anxiety was assessed using the Self-Rating Anxiety Scale (SAS) developed by Zung (24), a widely used instrument for evaluating the severity of anxiety symptoms. The scale consists of 20 items rated on a 4-point Likert scale, with several items reverse scored. Raw scores were multiplied by 1.25 to obtain standardized scores, with higher scores indicating greater severity of anxiety symptoms. A standardized score of 50 or higher was considered indicative of anxiety symptoms. In this study, SAS scores were used as a continuous measure of anxiety symptom severity rather than as a diagnostic indicator of anxiety disorder. The Cronbach's α coefficient for the SAS in this sample was 0.833, indicating good internal consistency.

#### Psychological resilience

Psychological resilience was assessed using the Connor–Davidson Resilience Scale (CD-RISC) (25),

The scale consists of 25 items rated on a 5-point Likert scale ranging from 1 (“not true at all”) to 5 (“true nearly all of the time”). The total score was calculated by summing all item scores, with higher scores indicating greater psychological resilience. In the present study, the overall CD-RISC demonstrated excellent internal consistency (Cronbach's α = 0.981). Cronbach's α coefficients for the tenacity, strength, and optimism subscales were 0.972, 0.825, and 0.953, respectively.

### Sample size estimation

The required sample size was estimated using the standard formula for cross-sectional studies: $n=Z_{1-\alpha /2}^{2}*P\;\left(1-P\right)/d$. Assuming a confidence level of 95% (*Z* = 1.96), an expected prevalence of 50%, and a margin of error equal to 10% of the estimated prevalence, the minimum required sample size was calculated to be approximately 385 participants. Considering potential non-response and invalid questionnaires, a substantially larger sample was recruited. The required sample size was estimated based on the recommended criteria for regression and mediation analyses, taking into account the number of predictors in the model. The final sample of 1,263 participants not only exceeded the minimum requirement but also provided sufficient statistical power for multivariable regression and mediation analyses, while allowing for potential invalid responses.

### Data quality control

Several measures were implemented to ensure data quality. Before the formal investigation, a pilot survey was conducted to improve questionnaire clarity and comprehensibility. During data collection, all questionnaire items were mandatory to minimize missing values, and duplicate submissions from the same IP address were automatically prevented. After data collection, questionnaires with implausibly short completion times, obvious response patterns, logical inconsistencies, or failure to meet the eligibility criteria were excluded from the final analysis. Data cleaning and verification were independently conducted by trained researchers before statistical analysis.

### Statistical analysis

All statistical analyses were performed using IBM SPSS Statistics version 27.0 (IBM Corp., Armonk, NY, USA) and PROCESS macro version 4.1 developed by Hayes. Continuous variables are presented as means ± standard deviations (SD), whereas categorical variables are summarized as frequencies and percentages. Internal consistency of the measurement scales was evaluated using Cronbach's alpha coefficients. Data normality was assessed using skewness and kurtosis statistics. Pearson correlation analysis was performed to examine bivariate associations among psychological resilience, job satisfaction, and anxiety symptoms. Hierarchical multiple linear regression analysis was subsequently conducted to determine the independent association between psychological resilience and anxiety symptoms after controlling for demographic characteristics. Additionally, interaction terms between psychological resilience and selected demographic characteristics (including sex, occupational category, and years of employment) were introduced into hierarchical regression models to examine whether the association between psychological resilience and anxiety symptoms differed across demographic subgroups.

Mediation analysis was performed using Hayes' PROCESS macro (Model 4) with 5,000 bootstrap resamples. An indirect effect was considered statistically significant when the corresponding 95% bootstrap confidence interval (CI) did not include zero. The magnitude of the indirect effect was additionally evaluated using the standardized mediation effect size (κ^2^), with values of 0.01, 0.09, and 0.25 indicating small, medium, and large effects, respectively. Potential common method bias was evaluated using Harman's single-factor test. Multicollinearity was assessed using variance inflation factors (VIFs), and residual independence was examined using the Durbin–Watson statistic. All statistical tests were two-tailed, and a *P*-value < 0.05 was considered statistically significant.

Additional interaction analyses were performed to examine two different types of interaction effects. First, moderation analyses were conducted to assess whether demographic characteristics modified the association between psychological resilience and anxiety symptoms. Interaction terms between psychological resilience and sex, occupational category, and years of employment were tested.

Second, to address potential joint effects of demographic characteristics, additional demographic interaction terms were created and entered into hierarchical regression models. Specifically, interactions between sex and occupational category, sex and years of employment, and occupational category and years of employment were examined after controlling for the corresponding main effects and other covariates.

## Results

### Participant characteristics

A total of 1,365 questionnaires were distributed, of which 1,263 were considered valid after data screening, yielding an effective response rate of 92.53%. Participant characteristics are presented in Table 1. Among the participants, 89.45% were female and 87.02% were nurses. Most participants were aged between 26 and 45 years (83.69%), had obtained a bachelor's degree or above (62.00%), and reported a monthly income of ≤ 6,000 RMB (87.25%). In addition, 65.24% had worked for fewer than 10 years. Detailed demographic characteristics are shown in Table 1.

**Table 1:** Positive rate of occupational anxiety and its distribution characteristics among healthcare professionals.

Characteristic	*N*	Proportion (%)	Number and detection rate of SAS tendency [*n* (%)]	χ^2^ -value	*P*-value
Gender				4.382	=0.036
Female	1130	89.45	260 (23.01)		
Male	133	10.55	20 (15.04)		
Age (years)				8.165	=0.043
≤ 25	95	7.52	12 (12.63)		
26 ~ 35	794	62.87	175 (22.04)		
36 ~ 45	263	20.82	70 (26.62)		
≥46	111	8.79	23 (20.72)		
Ethnicity				0.650	=0.420
Han	1227	97.15	274 (22.33)		
Minority	36	2.85	6 (16.67)		
Occupation				24.095	< 0.001
Doctor	164	12.98	12 (7.32)		
Nurse	1099	87.02	268 (24.39)		
Work experience (years)				26.521	< 0.001
≤ 5	401	31.75	58 (14.46)		
6 ~ 10	423	33.49	106 (25.06)		
11 ~ 19	299	23.67	89 (29.77)		
≥20	140	11.09	27 (19.29)		
Education level|				18.740	< 0.001
Technical secondary school	32	2.53	7 (21.88)		
College (diploma)	448	35.47	69 (15.40)		
Bachelor's degree or above	783	62.00	204 (26.05)		
Technical title				30.497	< 0.001
No title	360	28.50	48 (13.33)		
Junior	617	48.85	152 (24.64)		
Intermediate	246	19.48	75 (30.49)		
Senior associate or above	40	3.17	5 (12.50)		
Average monthly income (CNY)				10.851	=0.013
≤ 3,000	405	32.07	78 (19.26)		
3,001 ~ 6,000	697	55.18	162 (23.24)		
6,001 ~ 9,000	119	9.42	36 (30.25)		
≥9,001	42	3.33	4 (9.52)		
History of needlestick injury				1.018	=0.313
Yes	692	54.79	146 (21.10)		
No	571	45.21	134 (23.47)		
History of sharp object injury				3.340	=0.068
Yes	701	55.50	142 (20.26)		
No	562	44.50	138 (24.56)		
History of occupation-related infection				9.457	=0.002
Yes	17	1.35	9 (52.96)		
No	1246	98.65	271 (21.75)		
Daily sleep duration				13.505	< 0.001
< 8 h	991	78.46	242 (24.42)		
≥8 h	272	21.54	38 (13.97)		
Difficulty falling asleep				122.425	< 0.001
Yes	495	39.19	30 (6.06)		
No	768	60.81	250 (32.55)		
Sleep-disrupting events				203.232	< 0.001
Yes	621	49.17	242 ((38.97))		
No	651	50.83	38 (5.84)		

SAS refers to the Self-Rating Anxiety Scale. Corrected the numerical inconsistency for “History of Occupation-Related Infection” (Yes) from 52.47 to 52.94% (9/17). Corrected the formatting for “Sleep-Disrupting Events” (Yes) from “242 (38.97**)**” to “242 (38.97)”.

According to the Chinese normative criterion for the Self-rating Anxiety Scale (SAS), a standardized score of ≥50 indicates anxiety symptoms. In the present study, 280 participants were identified as having occupational anxiety, corresponding to an overall detection rate of 22.17%.

Significant differences in anxiety prevalence were observed across sex, age, occupation, years of employment, educational level, professional title, and monthly income (all *P* < 0.05). Participants with previous occupational infections, shorter sleep duration, sleep initiation difficulties, or sleep-disturbing events also exhibited significantly higher anxiety prevalence than their counterparts (all *P* < 0.05) (Table 1).

### Psychometric properties of the job satisfaction scale

The construct validity and internal consistency of the job satisfaction scale were evaluated before conducting the main analyses. The Kaiser–Meyer–Olkin (KMO) value was 0.964, and Bartlett's test of sphericity was statistically significant (χ^2^ = 24113.374, *P* < 0.001), indicating that the data were appropriate for factor analysis.

Exploratory factor analysis identified a single-factor structure, with one factor explaining 80.678% of the total variance. Because only one factor was extracted, factor rotation was not applicable. All 13 items demonstrated satisfactory factor representation within this unidimensional structure. The scale showed excellent internal consistency, with a Cronbach's alpha coefficient of 0.980.

### Descriptive statistics and reliability analysis of study variables

Descriptive statistics and psychometric properties of the study variables are presented in Table 2 (Panel A). The mean occupational anxiety score was 42.67 ± 7.55, whereas the mean psychological resilience score was 74.14 ± 20.78. The average scores for the three dimensions of psychological resilience were 26.95 ± 8.29 for tenacity, 5.76 ± 2.30 for strength, and 41.42 ± 13.23 for optimism, respectively. The mean job satisfaction score was 44.58 ± 11.61.

**Table 2:** Descriptive statistics, reliability, and correlation matrix of main study variables.

Panel A. Descriptive statistics and reliability analysis							
Variable	Items	*Mean*±*SD*	Minimum	Maximum	Skewness	Kurtosis	Cronbach's α
Anxiety symptoms	20	42.67± 7.55	25	75	0.57	0.87	0.833
Psychological resilience	25	74.14 ±20.78	25	121	−0.42	0.09	0.981
Tenacity	13	26.95 ± 8.29	8	40	−0.45	−0.18	0.972
Strength	8	5.76 ± 2.30	4	16	1.36	1.80	0.853
Optimism	4	41.42 ± 13.23	13	65	−0.20	−0.25	0.825
Job satisfaction	13	44.58 ± 11.61	13	65	−0.13	0.24	0.980
Panel B. Correlation matrix of primary study variables							
Variable		1		2		3	
1. Anxiety symptoms		1.000					
2. Psychological resilience		−0.209^**^		1.000			
3. Job satisfaction		−0.267^**^		0.317^**^		1.000	

^*^P < 0.05; ^**^P < 0.01 (two-tailed).

All measurement instruments demonstrated satisfactory internal consistency, with Cronbach's alpha coefficients ranging from 0.833 to 0.981. Skewness values ranged from −0.45 to 1.36, and kurtosis values ranged from −0.25 to 1.80, indicating that all continuous variables showed approximately normal distributions and met the assumptions required for subsequent Pearson correlation and hierarchical multiple linear regression analyses.

### Correlation analysis among study variables

Pearson correlation analysis demonstrated significant associations among the primary study variables (Table 2, Panel B). Occupational anxiety was significantly negatively correlated with psychological resilience (*r* = −0.209, *P* < 0.001) and job satisfaction (*r* = −0.267, *P* < 0.001). Psychological resilience was significantly positively correlated with job satisfaction (*r* = 0.317, *P* < 0.001). The correlation matrix including demographic and sleep-related covariates is presented in Supplementary Table S1. Among these variables, occupational anxiety was positively correlated with difficulty initiating sleep (*r* = 0.367, *P* < 0.001) and sleep-disturbing events (*r* = 0.476, *P* < 0.001), while it was negatively correlated with sleep duration (*r* = −0.170, *P* < 0.001). Other demographic characteristics showed relatively weak or non-significant correlations with the primary study variables. These findings demonstrate consistent bivariate associations among the main study variables and provide preliminary support for subsequent hierarchical regression and mediation analyses.

### Hierarchical multiple linear regression analysis

Hierarchical multiple linear regression analysis was conducted to examine the independent associations of psychological resilience and job satisfaction with anxiety symptom scores after adjusting for demographic and occupational characteristics. The results are presented in Table 3.

**Table 3:** Hierarchical linear regression analysis of Variables associated with anxiety symptom scores.

Variable	Model 1(β)	95%CI	Model 2(β)	95%CI	Model 3(β)	95%CI
Gender	−0.061	(−2.864, −0.139)	−0.052	(−2.620, 0.055)	−0.057	(−2.720, −0.089)
Age	−0.030	(−0.133, 0.070)	−0.008	(−0.108, 0.092)	−0.009	(−0.107, 0.089)
Years of work experience	0.033	(−0.476, 0.981)	0.026	(−0.518, 0.912)	0.028	(−0.492, 0.913)
Education level	0.086	(0.431, 1.982)	0.090	(0.498, 2.020)	0.089	(0.493, 1.989)
Occupation	0.082	(0.574, 3.126)	0.079	(0.511, 3.013)	0.087	(0.726, 3.189)
Professional title	0.013	(−0.611, 0.859)	0.016	(−0.569, 0.872)	0.018	(−0.538, 0.879)
Daily sleep duration	−0.076	(−8.311, −1.689)	−0.072	(−7.972, −1.478)	−0.069	(−7.723, −1.336)
Difficulty falling asleep	−0.082	(−2.464, −0.528)	−0.078	(-2.387,-0.488)	−0.064	(−2.115, −0.241)
Sleep-disturbing events	0.325	(4.206, 5.826)	0.316	(4.079, 5.669)	0.294	(3.752, 5.328)
Psychological resilience			−0.182	(−0.084, −0.048)	−0.127	(−0.065, −0.027)
Job satisfaction					−0.176	(−0.148, −0.081)
*R* ^2^	0.173		0.205		0.232	
Adjusted *R*^2^	0.167		0.199		0.226	
Δ*R*^2^			0.032		0.027	
*F*	29.108		32.368		34.448	
*P*	< 0.001		< 0.001		< 0.001	

Model 1 included demographic and occupational covariates, including sex, age, occupation, years of employment, monthly income, sleep duration, sleep initiation difficulties, and sleep-disturbing events. The model was statistically significant (*F* = 29.108, *P* < 0.001) and explained 17.3% of the variance in anxiety symptom scores (*R*^2^ = 0.173). Psychological resilience was subsequently entered into Model 2. Compared with Model 1, the explanatory power of the model significantly increased (Δ*R*^2^ = 0.032, *P* < 0.001). Psychological resilience remained independently associated with lower anxiety symptom scores (β = −0.182, 95% CI: −0.084 to −0.048, *P* < 0.001). Job satisfaction was further incorporated into Model 3. The inclusion of job satisfaction significantly improved the model (Δ*R*^2^ = 0.027, *P* < 0.001). Higher job satisfaction was independently associated with lower anxiety symptom scores (β = −0.176, 95% CI: −0.148 to −0.081, *P* < 0.001). Meanwhile, the standardized regression coefficient of psychological resilience was attenuated but remained statistically significant (β = −0.127, *P* < 0.001), suggesting that the association between psychological resilience and anxiety symptoms was partially explained by job satisfaction.

Several diagnostic analyses were performed to evaluate model robustness. Harman's single-factor test extracted five common factors, with the first factor accounting for 35.83% of the total variance, suggesting that substantial common method bias was unlikely. Variance inflation factors ranged from 1.007 to 3.697, indicating no evidence of multicollinearity. The Durbin–Watson statistic of the final regression model was 1.949, supporting the independence of residuals. Collectively, these results indicated that the assumptions of linear regression were adequately satisfied.

### Moderating effects of psychological resilience

Additional moderation analyses were conducted to examine whether demographic characteristics influenced the association between psychological resilience and anxiety symptoms. Interaction terms between psychological resilience and sex, occupational category, and years of employment were entered into the regression models.

### Interaction analyses between demographic characteristics and occupational factors

To further examine whether combinations of demographic and occupational characteristics were associated with differential levels of occupational anxiety symptoms, interaction analyses were conducted by introducing interaction terms between selected demographic variables. Specifically, the interaction effect between sex and occupational category was examined using multivariable linear regression models with occupational anxiety symptoms as the dependent variable. The interaction analysis was performed after adjusting for the same covariates included in the primary regression models.

No statistically significant interaction effect between sex and occupational category was observed after adjustment for potential confounders, suggesting that the association between demographic characteristics and occupational anxiety symptoms was relatively consistent across different occupational groups (Supplementary Table S2).

### Bootstrap analysis of the indirect association through job satisfaction

**Figure 1:**
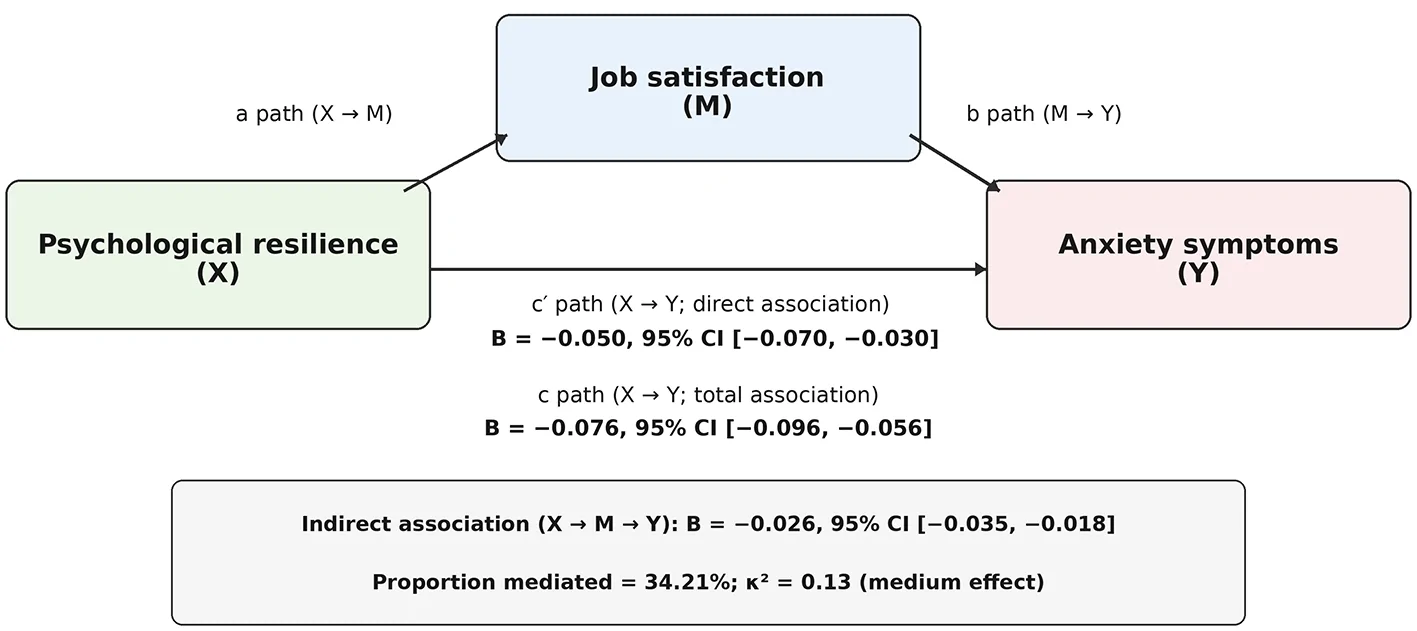
The tested mediation model of job satisfaction in the association between psychological resilience and anxiety symptoms. X = psychological resilience; M = job satisfaction; Y = anxiety symptoms. a path: association between X and M; b path: association between M and Y, controlling for X; c′ path: direct association between X and Y, controlling for M; c path: total association between X and Y. The indirect association was estimated using 5,000 bootstrap resamples; 95% bootstrap confidence intervals are presented.

A simple mediation model was tested to examine whether job satisfaction statistically accounted for part of the association between psychological resilience and anxiety symptoms (Figure 1). Psychological resilience was specified as the independent variable, job satisfaction as the mediator, and anxiety symptoms as the dependent variable. The mediation analysis was conducted using Hayes' PROCESS macro (Model 4) with 5,000 bootstrap resamples.

The total association between psychological resilience and anxiety symptoms was statistically significant (total effect = −0.076, SE = 0.010, 95% CI: −0.096 to −0.056). After including job satisfaction in the model, the direct association between psychological resilience and anxiety symptoms remained significant (direct effect = −0.050, SE = 0.010, 95% CI: −0.070 to −0.030). Bootstrap analysis indicated a statistically significant indirect association through job satisfaction (β = −0.026, 95% CI: −0.035 to −0.018), accounting for 34.21% of the total association between psychological resilience and anxiety symptoms. Given the cross-sectional design, this finding should be interpreted as a statistical indirect association rather than a causal mediation effect. In addition, the standardized mediation effect size was assessed using κ^2^. The κ^2^ value for the indirect effect was 0.13, suggesting a medium mediation effect. The results are presented in Table 4.

**Table 4:** Bootstrap analysis of the indirect association between psychological resilience and anxiety symptoms through job satisfaction.

Effect relationship	Effect size	SE	95%*CI*	Percentage of total association (%)
Total effect	−0.076	0.010	−0.096–−0.056	
Direct effect	−0.050	0.010	−0.070–−0.030	65.79
Indirect effect	−0.026	0.004	−0.035–−0.018	34.21

Total effect = direct effect + indirect effect. The indirect effect represents the extent to which the association between psychological resilience and anxiety symptoms is statistically accounted for by job satisfaction. In practical terms, this pathway indicates that healthcare workers with higher psychological resilience tended to report greater job satisfaction, which was in turn associated with fewer anxiety symptoms. Bootstrap confidence intervals were based on 5,000 resamples. An indirect effect was considered statistically significant when the 95% confidence interval did not include zero. κ^2^ represents the standardized mediation effect size, with values of 0.01, 0.09, and 0.25 indicating small, medium, and large effects, respectively.

## Discussion

### Main findings

This multicenter cross-sectional study of 1,263 healthcare workers from 10 private hospitals found that psychological resilience and job satisfaction were each significantly associated with anxiety symptoms—resilience linked to lower anxiety scores, and job satisfaction positively associated with resilience while negatively associated with anxiety. Beyond demographic and occupational characteristics, both factors explained additional variance in anxiety symptoms. Moreover, bootstrap analysis indicated that job satisfaction statistically accounted for part of the association between psychological resilience and anxiety symptoms. Together, these findings underscore the potential importance of both personal psychological resources and workplace-related factors in understanding and supporting healthcare workers' mental wellbeing in private hospital settings (26, 27).

### Association between psychological resilience and anxiety symptoms

The present study found that psychological resilience was significantly associated with lower anxiety symptom scores among healthcare workers. This finding is consistent with previous research suggesting that resilience represents an important psychological resource related to better mental health outcomes (28–30). According to the Job Demands–Resources (JD-R) model, personal resources may influence how individuals perceive and respond to occupational demands (17, 18). Healthcare workers with higher levels of resilience may possess greater psychological capacity to adapt to workplace stressors, which may be associated with reduced psychological distress.

Importantly, our findings should be interpreted as an association rather than a causal effect. Because of the cross-sectional design, it remains unclear whether higher resilience leads to fewer anxiety symptoms or whether individuals with fewer psychological difficulties are more likely to report higher resilience. Longitudinal studies are needed to further clarify the temporal relationship between resilience and anxiety symptoms.

### Role of job satisfaction in the association between resilience and anxiety symptoms

Job satisfaction was negatively associated with anxiety symptom scores and statistically accounted for part of the association between psychological resilience and anxiety symptoms. This finding suggests that workplace-related experiences may represent one potential explanatory factor linking individual psychological resources with mental health outcomes (31, 32).

From the perspective of the JD-R model, job satisfaction reflects a positive work-related outcome associated with motivational processes. In addition, Self-Determination Theory emphasizes that the fulfillment of psychological needs, such as competence, autonomy, and relatedness, may contribute to positive emotional experiences in the workplace. Therefore, healthcare workers who perceive greater satisfaction with their work may experience more positive occupational functioning and psychological wellbeing.

However, the observed indirect association should not be interpreted as evidence of a causal mediation mechanism. Future longitudinal and intervention studies are required to determine whether improvements in job satisfaction can contribute to changes in anxiety symptoms over time.

### Theoretical implications

This study contributes to the existing literature in several ways. First, by focusing on healthcare workers in private hospitals, this study extends previous research that has predominantly examined psychological wellbeing among workers in public healthcare institutions. Although private hospitals may differ from public institutions in organizational structures and employment environments, healthcare workers in both settings face substantial occupational demands that require effective psychological resources (9–11).

Second, this study highlights the importance of considering both individual psychological resources and workplace-related experiences when examining healthcare workers' mental health. Rather than viewing anxiety symptoms as solely determined by external occupational stressors, our findings suggest that personal resilience and job-related experiences may jointly contribute to differences in psychological wellbeing (33, 34).

In addition, the absence of significant demographic interaction effects suggests that the associations between psychological resilience, job satisfaction, and anxiety symptoms were relatively stable across different demographic subgroups. This finding further supports the general applicability of the proposed framework among healthcare workers in private hospital settings.

### Practical implications

The findings may have practical implications for healthcare organizations seeking to promote healthcare workers' psychological wellbeing. First, healthcare organizations may consider paying attention to both individual psychological resources and workplace-related experiences when designing future mental health promotion strategies. Second, improving job satisfaction may represent another potential direction, including enhancing organizational support, improving communication mechanisms, and creating supportive working environments.

Importantly, interventions should not focus solely on individual resilience while overlooking organizational factors. A comprehensive approach that considers both personal resources and workplace conditions may provide a more sustainable strategy for promoting healthcare workers' mental health.

### Strengths and limitations

This study has several strengths. First, it included a relatively large sample of healthcare workers from multiple private hospitals, providing valuable evidence regarding psychological wellbeing in an understudied healthcare setting. Second, the study simultaneously examined individual psychological resources and workplace-related factors, providing a more comprehensive perspective on factors associated with anxiety symptoms. Third, the use of bootstrap analysis allowed further examination of the potential indirect association involving job satisfaction while maintaining appropriate caution regarding causal interpretation.

Several limitations should be acknowledged when interpreting the present findings. First, the cross-sectional design precludes any causal inferences regarding the relationships among psychological resilience, job satisfaction, and anxiety symptoms. Future longitudinal studies are warranted to examine temporal dynamics and potential intervention effects. Second, participants were recruited from 10 private hospitals in a single city using convenience sampling. Although the sample size is relatively large, the generalizability of the findings to healthcare workers in other regions, public hospital settings, or different healthcare systems may be limited. Furthermore, detailed hospital-level information—such as institutional size, organizational characteristics, and distribution patterns—was unavailable, which precluded further examination of potential clustering effects. Third, all primary variables were assessed via self-reported questionnaires, raising the possibility of reporting bias. Although procedural and statistical remedies were implemented to mitigate common method variance, future research incorporating objective indicators or multi-source data would strengthen the robustness of conclusions. Fourth, the job satisfaction scale employed in this study was developed on the basis of prior literature and expert consultation, and it demonstrated excellent internal consistency and a clear unidimensional structure in our sample. Nevertheless, independent validation using confirmatory factor analysis in other populations is still needed. Finally, several occupational and organizational factors that may influence anxiety symptoms—including workload, working hours, night-shift exposure, and perceived organizational support—were not collected in this survey. As a result, residual confounding cannot be entirely ruled out, and future studies should consider incorporating these contextual variables to better disentangle the multifactorial nature of healthcare workers' psychological wellbeing.

## Conclusions

This study identified significant associations among psychological resilience, job satisfaction, and anxiety symptoms in healthcare workers, with higher levels of both resilience and job satisfaction linked to lower anxiety scores, and job satisfaction statistically accounting for part of the resilience–anxiety relationship. Nevertheless, these cross-sectional findings require confirmation through longitudinal designs to establish temporal precedence. Moreover, although statistical tests did not reveal substantial common method bias, the reliance on self-reported questionnaires administered at a single time point means that residual reporting bias cannot be entirely ruled out, underscoring the need for future research employing multi-wave or multi-source data collection strategies.

## Data Availability

The raw data supporting the conclusions of this article will be made available by the authors, without undue reservation.
